# Supplementary figures and images for: Cellular stress modulates severity of the inflammatory response in lungs via cell surface BiP
Source: Front Immunol. 2022 Nov 18;13:1054962. doi: 10.3389/fimmu.2022.1054962 (PMC9716134; doi:10.3389/fimmu.2022.1054962)

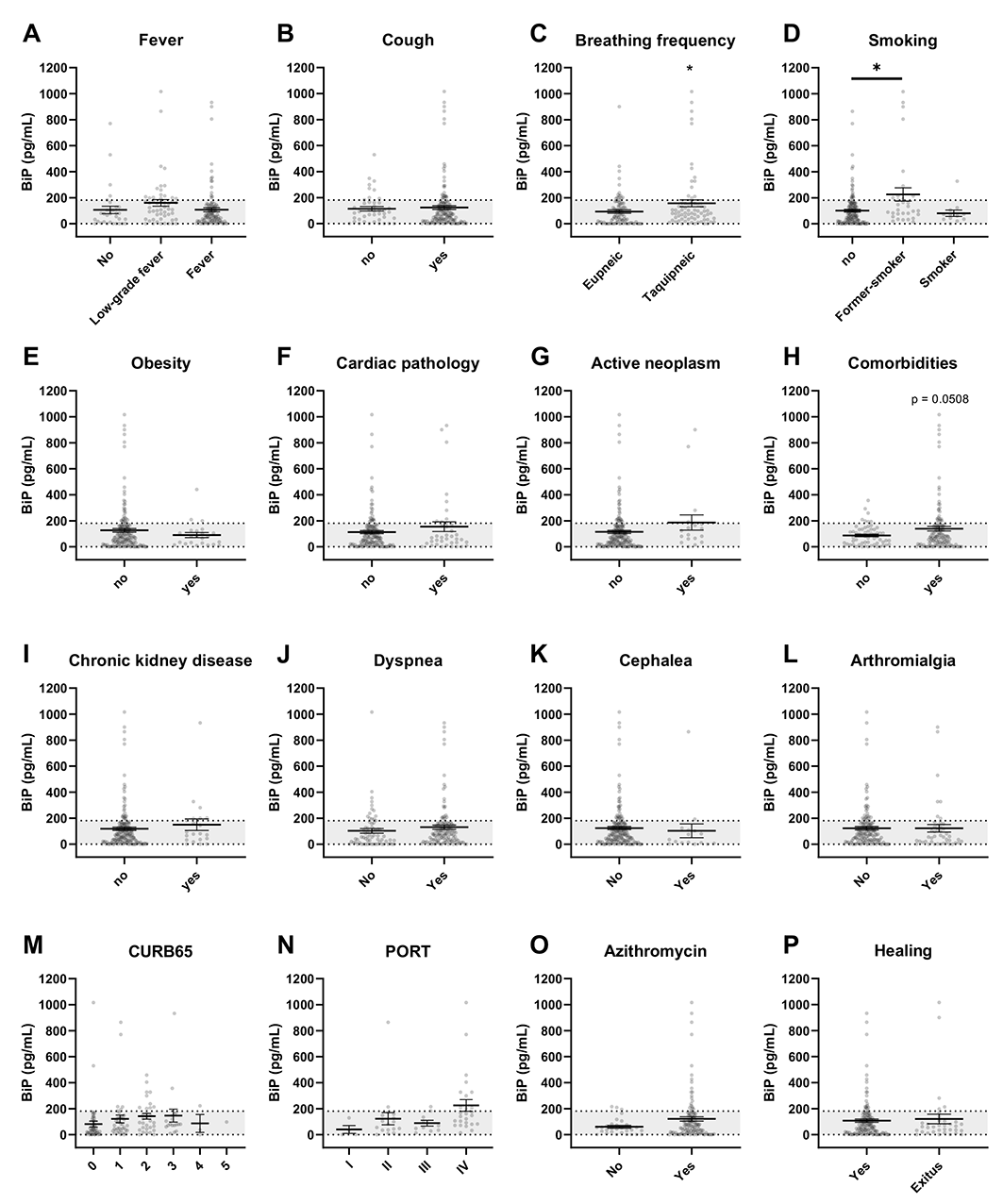

Supplement: Supplementary Figure 1 — (A–P) Serum BiP levels classified by group of patients/donors. Black lines and whiskers denote the mean ± SEM of every data set. Green areas were defined between 5th and 95th percentiles of healthy donor’s data set as normal BiP levels in serum (0 and 181 pg/mL, respectively). *P < 0.05, **P < 0.01, ***P < 0.001 indicate statistical significant differences between samples for a Two-Tailed unpaired t-Test (A–C, E–L, O,P) and One-Way ANOVA with a Tukey’s multiple comparisons test (D, M, N). [file Image_1.tif]

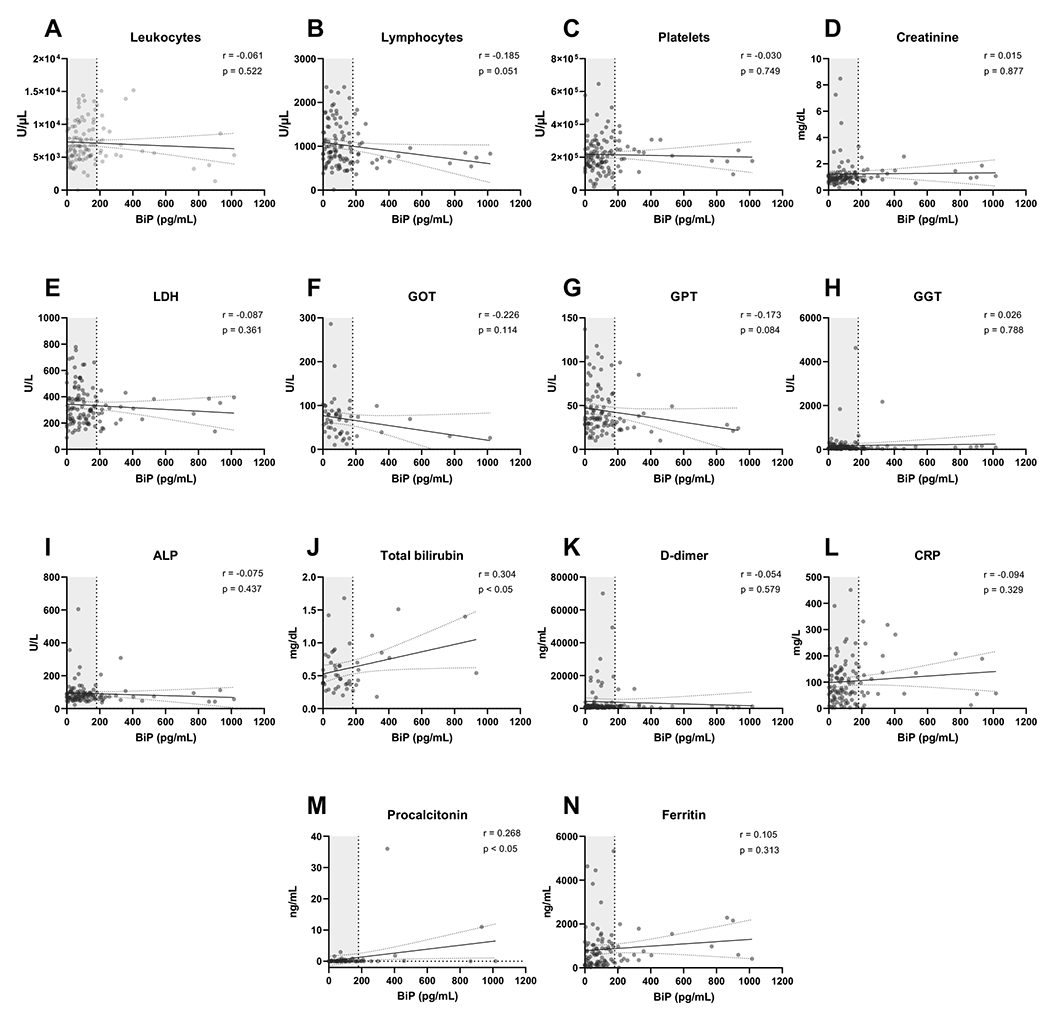

Supplement: Supplementary Figure 2 — (A–N) Scatter plots showing the correlation between BiP levels versus different hematological and biochemical parameter levels in COVID-19 patient’s blood serum tested by Pearson’s correlation coefficient (r and its related P-value). Bold line shows the linear regression between the two variables and dotted lines denote the 95% confidence interval. [file Image_2.tif]

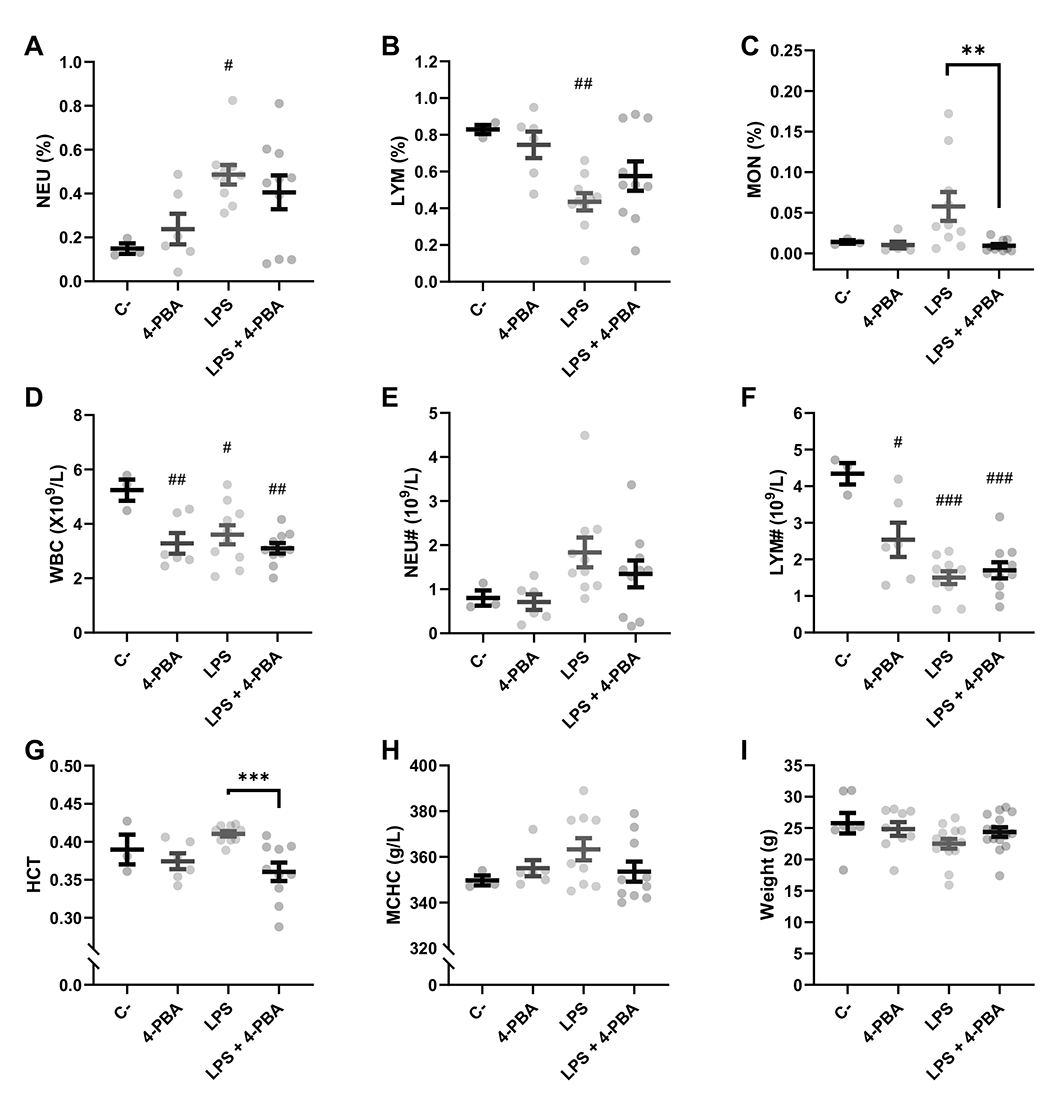

Supplement: Supplementary Figure 3 — (A–H) Hematological analyses: percentages of neutrophils (A), lymphocytes (B) and monocytes (C), total numbers of white blood cells (WBC) (D), total numbers of neutrophils (E) and total numbers of lymphocytes (F), hematocrit (HCT) (G) and mean corpuscular hemoglobin concentration (MCHC) (H) in total blood obtained from mice challenged with LPS without 4-PBA treatment (LPS, n=10, graphed in red) and with 4-PBA treatment (LPS + 4-PBA, n=10, graphed in blue). Groups of unchallenged mice without 4-PBA (C-, n=3, graphed in black) and with 4-PBA treatment (4-PBA, n=6; graphed in green) were also evaluated. (I) Animal’s weight for every experimental group. Colored lines and whiskers denote mean ± SEM for every data set. Hash marks indicate significant difference versus C- (# P < 0.05, ## P < 0.01, ### P < 0.001) and asterisks between samples linked by a line (*P < 0.05, **P < 0.01, ***P < 0.001) for a Two-way ANOVA followed by Tukey’s post-hoc test. [file Image_3.tif]

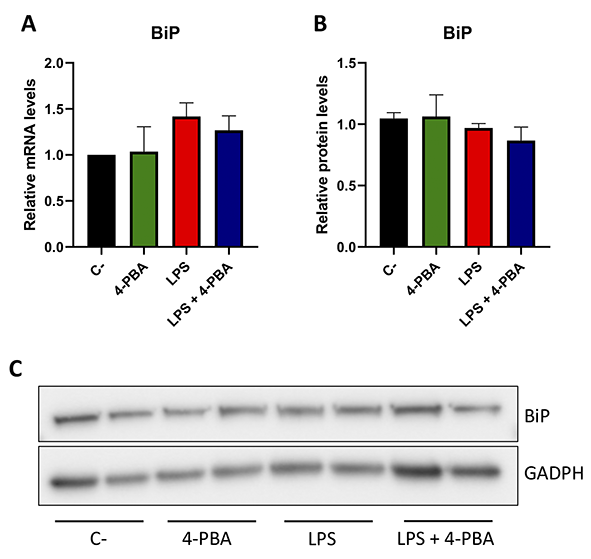

Supplement: Supplementary Figure 4 — (A) Quantitative gene expression of Hspa5 (BIP) measured by RT-qPCR. (B, C) Total (pan) levels of BiP protein in lung tissues; western blot (B) and corresponding quantification of protein levels representation (C). No statistical significance was found among the represented conditions. [file Image_4.tif]

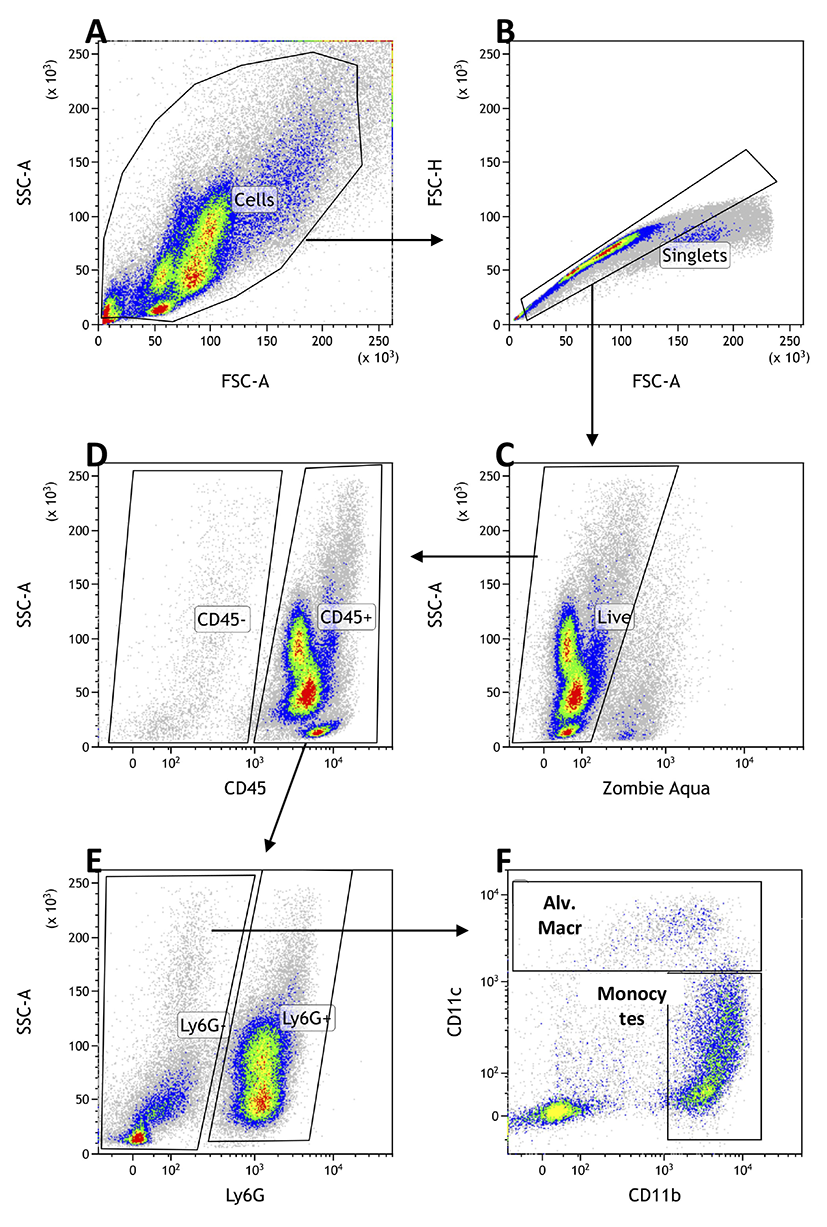

Supplement: Supplementary Figure 5 — Flow cytometry gating strategy. (A) Representative scatter plots showing FSC-A x SSC-A gating to exclude debris based on size and granularity; (B) FSC-A x FSC-H to exclude doublets. (C) Zombie Aqua™ fixable viability marker to identify live cells (negative for the marker. (D) Staining with CD45 to identify hematopoietic cell linages. (E) Ly6G was used to identify neutrophils (as Ly6G+). (F) Among the Ly6G- cells, CD11b x CD11c was used to identify alveolar macrophages and dendritic cells population (as CD11c+ with variable levels of CD11b) and monocytes as long as other myeloid subsets (as CD11b+ CD11c-/low). Gates for all viability marker and all antibodies were determined using respective fluorescence minus one (FMO) control. [file Image_5.tif]

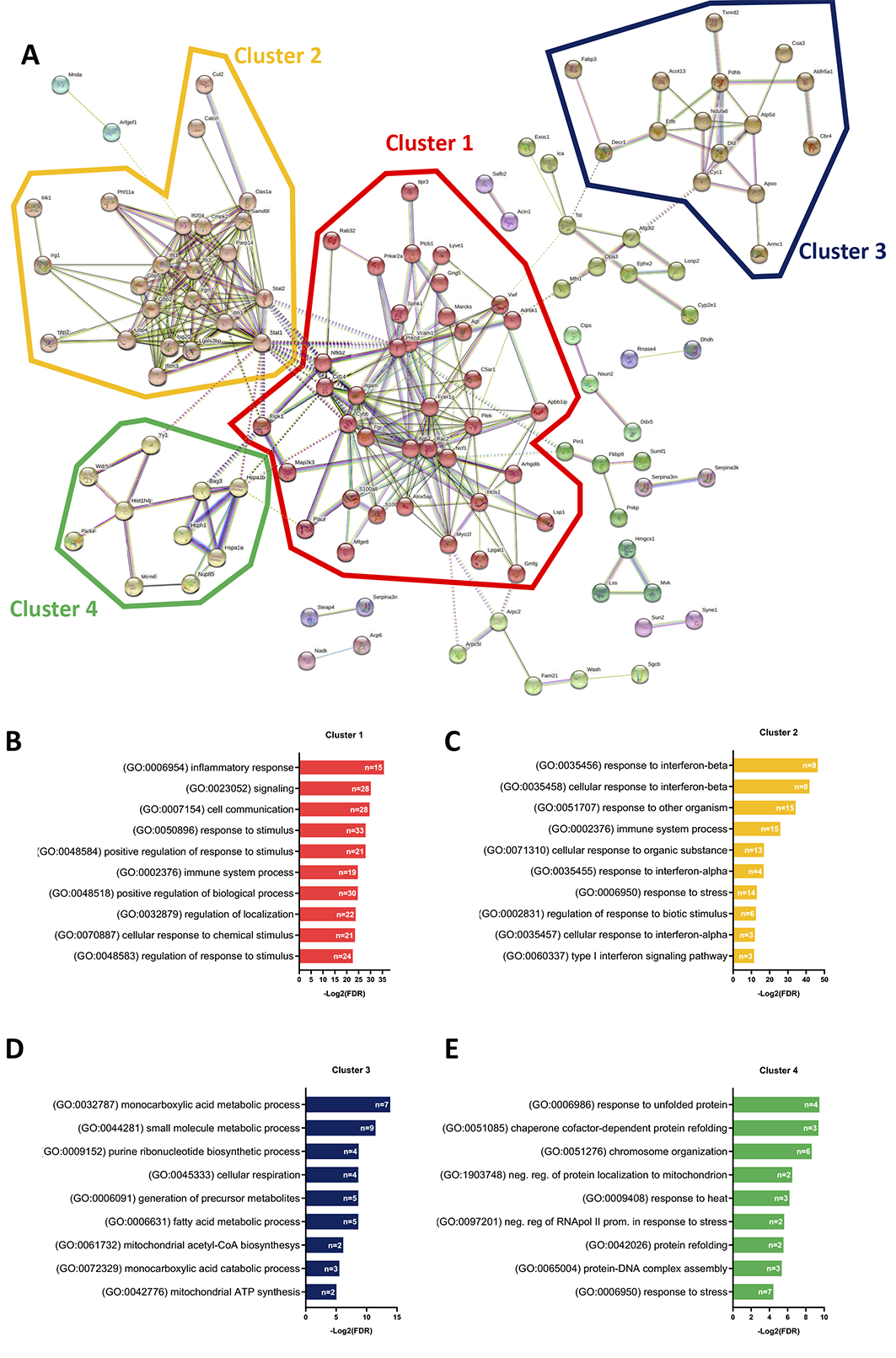

Supplement: Supplementary Figure 6 — (A) StringDB network (without inclusion of Hspa5) showing the associations between proteins differentially expressed in response to LPS challenge in mice lungs forming 4 principal clusters detected by an unsupervised Markov Cluster Algorithm (MCL). (B–E) Bar plots showing the Top-10 enriched Biological Processes associated with every cluster ordered by False Discovery Rate. Single bars indicate the number of proteins associated with every GOterm. [file Image_6.tif]

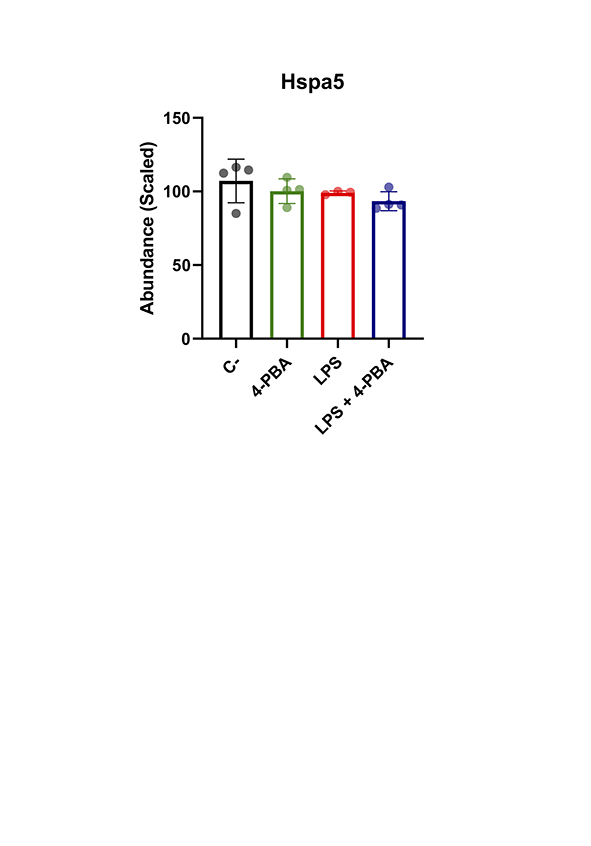

Supplement: Supplementary Figure 7 — BiP Levels measured by proteomic analysis in lung tissues showing the mean ± SD of the scaled abundances. There were no statistically significant differences between samples for a One-Way ANOVA with a Tukey’s multiple comparisons test (n=4 for C-, 4-PBA and LPS + 4-PBA groups; n=3 for LPS group). [file Image_7.tif]
