# Supplementary material for: Cellular stress modulates severity of the inflammatory response in lungs via cell surface BiP
Source: Front Immunol. 2022 Nov 18;13:1054962. doi: 10.3389/fimmu.2022.1054962 (PMC9716134; doi:10.3389/fimmu.2022.1054962)
Supplement: Supplementary file 8 [file DataSheet_1.pdf]

Supplementary Table 1: Pneumonia severity categories

Severity	Oxygen requirement	Parametric values
No pneumonia	No	- Saturation >94%<br>- Eupneic *
Mild severity	Low Flow	- Saturation <94%<br>- Eupneic *
Moderate severity	Low Flow	- Saturation 90%-94%<br>- Possible Tachypnea**<br>- Risk factors identified
High severity	High Flow<br>Noninvasive mechanic ventilation	- Saturation <90%<br>- Possible Tachypnea**<br>- Risk factors identified
Very high severity	Invasive mechanic ventilation	- Saturation <90%<br>- Tachypnea<br>- Risk factors identified

* Eupneic <22RPM
** Tachypnea (22 O > 22 RPM)
